# Implication of NOD1 and NOD2 for the Differentiation of Multipotent Mesenchymal Stem Cells Derived from Human Umbilical Cord Blood

**DOI:** 10.1371/journal.pone.0015369

**Published:** 2010-10-22

**Authors:** Hyung-Sik Kim, Tae-Hoon Shin, Se-Ran Yang, Min-Soo Seo, Dong-Jae Kim, Soo-Kyung Kang, Jong-Hwan Park, Kyung-Sun Kang

**Affiliations:** 1 Adult Stem Cell Research Center, College of Veterinary Medicine, Seoul National University, Seoul, South Korea; 2 Department of Pathology, University of Michigan Medical School, Ann Arbor, Michigan, United States of America; 3 Department of Veterinary Biotechnology, College of Veterinary Medicine, Seoul National University, Seoul, South Korea; 4 Department of Biochemistry, College of Medicine, Konyang University, Daejeon, South Korea; Charité-University Medicine Berlin, Germany

## Abstract

Toll-like receptors (TLRs) and Nod-like receptors (NLRs) are known to trigger an innate immune response against microbial infection. Although studies suggest that activation of TLRs modulate the function of mesenchymal stem cells (MSCs), little is known about the role of NLRs on the MSC function. In this study, we investigated whether NOD1 and NOD2 regulate the functions of human umbilical cord blood-derived MSCs (hUCB-MSCs). The genes of TLR2, TLR4, NOD1, and NOD2 were expressed in hUCB-MSCs. Stimulation with each agonist (Pam_3_CSK_4_ for TLR2, LPS for TLR4, Tri-DAP for NOD1, and MDP for NOD2) led to IL-8 production in hUCB-MSC, suggesting the expressed receptors are functional in hUCB-MSC. CCK-8 assay revealed that none of agonist influenced proliferation of hUCB-MSCs. We next examined whether TLR and NLR agonists affect osteogenic-, adipogenic-, and chondrogenic differentiation of hUCB-MSCs. Pam_3_CSK_4_ and Tri-DAP strongly enhanced osteogenic differentiation and ERK phosphorylation in hUCB-MSCs, and LPS and MDP also slightly did. Treatment of U0126 (MEK1/2 inhibitor) restored osteogenic differentiation enhanced by Pam_3_CSK_4_. Tri-DAP and MDP inhibited adipogenic differentiation of hUCB-MSCs, but Pam_3_CSK_4_ and LPS did not. On chondrogenic differentiation, all TLR and NLR agonists could promote chondrogenesis of hUCB-MSCs with difference in the ability. Our findings suggest that NOD1 and NOD2 as well as TLRs are involved in regulating the differentiation of MSCs.

## Introduction

Toll-like receptors (TLRs) are type I transmembrane proteins and composed of extracellular leucine rich repeats (LRRs) domain that are responsible for recognition of pathogen-associated molecular patterns (PAMPs) and intracellular Toll/IL-1R (TIR) domain, which is essential for downstream signaling. TLRs recognize microbial molecules including lipoprotein (TLR2), LPS (TLR4), flagellin (TLR5), dsRNA (TLR3), ssRNA (TLR7/8), and CpG DNA motif (TLR9), and subsequently activate NF-κB and MAPK to trigger inflammatory process [1]. In addition to microbial molecules, a variety of endogenous agonists such as heat shock proteins, high mobility group box 1 (HMGB1), hyaluronan fragments, heparin sulphate, and fibronectin are recognized by TLR2 or TLR4 [2].

As another PRRs family, Nod-like receptors (NLRs) are intracellular, cytoplasmic sensor for microbial components and danger signals [3], [4]. There are 23 NLR family members in humans and at least 34 genes in mice [4]. NLRs are expressed in nonimmune cells including epithelial cells and mesothelial cells as well as immune cells. As first identified NLRs, NOD1 and NOD2 consist of N-terminal caspase recruitment domain (CARD), intermediate nucleotide-binding oligomerization domain (NOD), and C-terminal leucine-rich repeats (LRRs) domain. NOD1 and NOD2 recognize peptidoglycan (PGN) derivatives, *meso*-diaminopimelic acid (*meso*-DAP) and muramyl dipeptide (MDP), respectively. After stimulation by their specific bacterial molecules, NOD1 and NOD2 associate with an adaptor molecule, RICK/Rip2/CARDIAK, through CARD-CARD interaction, which leads to activation of NF-κB and MAPK and induction of numerous genes involved in inflammatory process [5], [6].

Mesenchymal stem cells (MSCs) are multipotent adult progenitor cells that can differentiate to various cell types including osteoblasts, adipocytes, chondrocytes, cardiomyocytes, fibroblasts, and endothelial cells [7], [8], [9]. MSCs are thought to be excellent candidate tools for the field of regenerative medicine, because of their differentiation potential. In addition, MSCs were found to suppress proliferation, differentiation, and activation of immune cells including T cells, B cells, NK cells, and dendritic cells [10], [11].

The isolation of MSCs have been described in several species and from different tissues, including bone marrow (BM), peripheral blood, adult fat, umbilical cord blood (UCB) and skeletal muscle. Among MSCs, the most commonly used for clinical purposes are isolated from BM or adipose tissue. Stem cells from UCB have many advantages because of the immature nature of newborn cells compared to adult cells. In our previous study, we showed that hUCB-MSCs express OCT4A and REX1, a well-known transcription factors that are characteristic markers of pluripotent stem cells, and REX1 expression in hUCB-MSCs was nearly five-fold greater than in hBM-MSCs [12], [13]. Moreover, hUCB-MSCs provide no ethical barriers for basic studies and clinical applications [14], [15].

Recent studies showed that TLRs regulate MSCs functions such as proliferation, differentiation, migration, and immunomodulation [16], [17], [18]. Although van den Berk *et al* [19], recently reported the involvement of TLRs in the regulation of the functions of cord blood MSCs, most studies have focused on BM- or adipose tissue-derived MSCs (ASC). In addition, little is known about the role of NLRs on MSCs functions. This study was performed to clarify the role of NOD1 and NOD2 on the proliferation and differentiation of hUCB-MSCs. We show here that NOD1 and NOD2 as well as TLRs are involved in regulating the differentiation of hUCB-MSC.

## Materials and Methods

### Isolation and culture of hUCB–MSCs

The UCB samples were obtained from the umbilical vein immediately after delivery, with the written informed consent of the mother approved by the Boramae Hospital Institutional Review Board (IRB) and the Seoul National University IRB(IRB No. 0603/001-002-07C1). The UCB samples were mixed with the Hetasep solution (StemCell Technologies, Vancouver, Canada) at a ratio of 5∶1, and then incubated at room temperature to deplete erythrocyte counts. The supernatant was carefully collected and mononuclear cells were obtained using Ficoll density-gradient centrifugation at 2,500 rpm for 20 min. The cells were washed twice in PBS. Cells were seeded at a density of 2×10^5^ to 2×10^6^ cells/cm^2^ on plates in growth media consisted of D-media (Formula No. 78-5470EF, Gibco BRL) containing EGM-2 SingleQuot and 10% fetal bovine serum (Gibco BRL). After 3 days, non-adherent cells were removed. The adherent cells formed colonies and grew rapidly, exhibiting spindle-shaped morphology.

### Reagents

Ultrapure LPS (*E. coli* O111:B4), Pam_3_CSK_4_, and Tri-DAP were purchased from Invivogen (San Diego, CA, USA). MDP [Ac-(6-O-stearoyl)-muramyl-Ala-D-Glu-NH_2_] was from Bachem (Bubendorf, Switzerland). MEK1/2 Inhibitor U0126 was from Promega (Madison, WI, USA).

### Flow cytometric analysis

hUCB-MSCs(1×10^6^/ml) were stained with FITC- or PE- conjugated antibodies specific for human CD14, CD29, CD31, CD33, CD34, CD44, CD45, CD73, CD90, CD105, CD133, and HLA-DR. Non-specific isotype-matched antibodies served as controls. All the antibodies were purchased from BD Bioscience, and flow cytometry analysis was performed on a FACSCaliber using the Cell Quest software (Becton Dickinson, Franklin Lakes, NJ, USA).

### RNA extraction and RT-PCR

Total RNA was extracted from hUCB-MSCs by using Easy-spin total RNA extraction kit (Intron Biotechnology, Seongnam, Korea) according to the manufacture's protocol. cDNA was prepared from 1 µg of total RNA by using Superscript III reverse transcriptase (Invitrogen, Carlsbad, CA, USA) and oligo (dT) primers (Invitrogen). The primer sets used were as follows;

TLR2, F: 5′- GATGCCTACTGGGTGGAGAA-3′, R: 5′- CGCAGCTCTCAGATTTACCC-3′

TLR4, F: 5′- ACAGAAGCTGGTGGCTGTG-3′, R: 5′- TCTTTAAATGCACCTGGTTGG-3′

NOD1, F: 5′- CCACTTCACAGCTGGAGACA-3′, R: 5′- TGAGTGGAAGCAGCATTTTG-3′

NOD2, F: 5′- GAATGTTGGGCACCTCAAGT-3′, R: 5′- CAAGGAGCTTAGCCATGGAG-3′

Rip2, F: 5′- CCATTGAGATTTCGCATCCT-3′, R: 5′- ATGCGCCACTTTGATAAACC-3′

RPL13A, F: 5′- CATCGTGGCTAAACAGGTAC-3′, R: 5′- GCACGACCTTGAGGGCAGCC-3′

The PCR condition consisted of an initial denaturation at 95°C for 3 min; 30 cycles of 94°C for 30 sec, 60°C for 30 sec and 72°C for 1 min; a final extension at 72°C for 10 min. The PCR products were separated on a 1.5% agarose gel, visualized, and photographed using a gel documentation system.

### Cytokine production

Briefly, hUCB-MSCs (2×10^4^/well) were seeded in MSC medium supplemented with 2% FBS in 96-well plate. Twenty-four hours later, the cells were treated with various doses of Pam_3_CSK_4_, LPS, Tri-DAP, and MDP and incubated for additional 24 h. Culture supernatant was collected, centrifuged, filtered through 0.2 µm filter and IL-8 concentration was measured using commercial ELISA kit (R&D Systems, Minneapolis, MN, USA) according to manufacturer's protocol.

### hUCB-MSC proliferation

The cells were seeded at 2 × 10^3^/well in 96-well plates in MSC medium supplemented with 2% FBS. Twenty-four hours later, the cells were treated with Pam3CSK_4_, LPS, Tri-DAP, and MDP at 10 µg/ml concentration and incubated for 4 days. Proliferation was determined by Cell Counting Kit-8 (Dojindo Molecular Technologies, Rockville, MD, USA) according to manufacturer's instruction.

### hUCB-MSC differentiation

#### Osteogenic differentiation

The cells were incubated in conditioned media containing DMEM low glucose medium, 10% FBS, 0.1 µM dexamethasone, 10 mM beta-glycerophosphate, and 50 µM ascorbate at the absence or presence of TLR and NLR agonists. The cells were grown for 2 weeks, with medium replacement twice a week. Osteogenesis was detected by Alizarin Red staining. Photographs were taken and optical density was measured at 570 nm.

#### Adipogenic differentiation

The cells were incubated in conditioned media containing DMEM low glucose medium, 10% FBS, 1 M dexamethasone, 10 µg/ml insulin, 0.5 mM 3-isobutyl-1-methylxanthine, and 0.2 mM indomethacin at the absence or presence TLR and NLR agonists. The cells were grown for 3 weeks, with media replacement twice a week. Adiopogenesis was detected by Oil red O staining. Photographs were taken and optical density was measured at 500 nm.

#### Chondrogenic differentiation

2×10^5^ cells were seeded in 15-mL polypropylene tube and centrifuged to a pellets. The pellets were cultured in 1 ml of chondrogenic medium that contained 10% FBS and 500 ng/ml bone morphogenetic protein-2(BMP-2; R&D Systems) for 3 weeks. The chondrogenic differentiation medium was replaced twice a week. The pellets were embedded in paraffin and cut into 3 µm sections. For histological evaluation, the sections were stained with toluidine blue following general procedures.

### Western blot

The cells were stimulated with agonists, harvested, and lysed in buffer containing 1% Nonidet-P40 supplemented with complete protease inhibitor ‘cocktail’ (Roche) and 2 mM dithiothreitol. Lysates were resolved by 12% SDS-PAGE, transferred to nitrocellulose membranes, and immunoblotted with primary antibodies such as regular- and phopho-ERK (Cell signaling, Beverly, MA, USA) and GAPDH (Santa Cruz biotechnology, Santa Cruz, CA, USA). After immunoblotting with secondary antibodies, proteins were detected with enhanced chemiluminescence (ECL) reagent (Intron Biotechnology).

### Statistical analysis

The differences in mean values among different groups were tested, and the values were expressed as mean ± SD. All of the statistical calculations were calculated by one-way ANOVA followed by Bonferroni post-hoc test for multigroup comparisons (StatView 5.0; SAS Institute, Cary, NC). Statistical significance is indicated in the figure legends.

## Results

### TLR2, TLR4, NOD1, and NOD2 were functionally expressed in hUCB-MSCs

**Figure 1:**
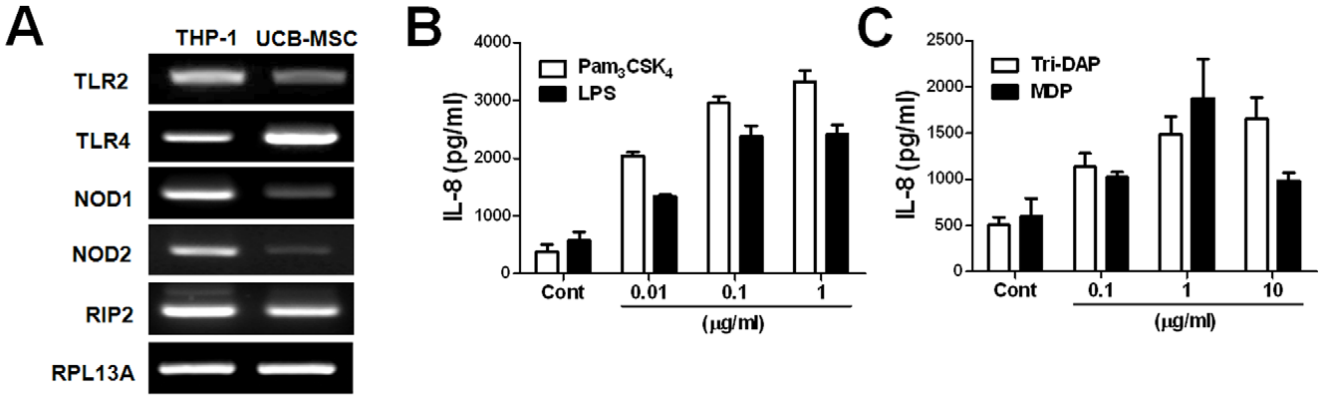
TLRs and NLRs were functionally expressed in hUCB-MSCs. mRNA expressions of TLR2, TLR4, NOD1, NOD2, and Rip2 were determined by RT-PCR in hUCB-MSCs (A). The cells were treated with Pam_3_CSK_4_, LPS (B), Tri-DAP, and MDP (C) in a dose-dependent manner for 24 h and IL-8 production was determined using a commercial ELISA kit (B and C).

To verify the stem cell phenotypic markers of hUCB-MSCs using flow cytometry, we observed that hUCB-MSCs were negative for CD14, CD31, CD33, CD34, CD45, CD133 and HLA-DR expression but positive for CD29, CD44, CD73, CD90 and CD105 (data not shown). The gene expression of TLR2, TLR4, NOD1, and NOD2 in hUCB-MSCs was examined by RT-PCR. A human monocytic leukemia cell line, THP-1 cells were used as positive control. All receptors tested were expressed in both THP-1 cells and hUCB-MSCs (Fig. 1A). TLR4 was expressed more strongly in hUCB-MSCs than in THP-1 cells, whereas the gene expression of TLR2, NOD1, and NOD2 was weaker in UCB-MSC (Fig. 1A). Rip2, the adaptor protein of NOD1 and NOD2, was also apparently expressed in hUCB-MSCs (Fig. 1A). To evaluate the functionality of the receptors, we examined IL-8 production by hUCB-MSCs in response to their specific agonists. Stimulation by Pam_3_CSK_4_ (TLR2 agonist), LPS (TLR4), Tri-DAP (NOD1), and MDP (NOD2) led to increased production of IL-8 in hUCB-MSCs in a dose-dependent manner (Fig. 1B and C). These findings indicate that NOD1 and NOD2, as well as TLR2 and TLR4, are expressed in hUCB-MSCs and can respond to their specific agonists.

### Activation of TLRs and NLRs did not influence the proliferation of hUCB-MSCs

**Figure 2:**
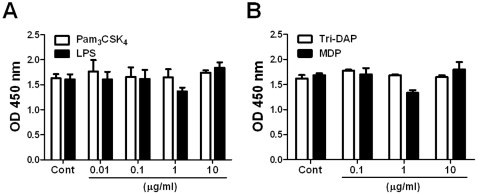
Activation of TLRs and NLRs did not influence the proliferation of hUCB-MSCs. hUCB-MSCs were treated with various doses of Pam_3_CSK_4_, LPS (A), Tri-DAP, and MDP (B) for 4 days and cell proliferation was determined by CCK-8 kit.

TLRs have been found to promote the proliferation of several types of MSC [17], [20], [21]. To examine whether TLR and NLR activation influence the proliferation of hUCB-MSC, the cells were incubated at the absence or presence of each agonist (Pam_3_CSK_4_, LPS, Tri-DAP, and MDP) for 4 days and cell proliferation was determined by CCK-8 analysis. Results showed that none of agonists influenced the proliferation of hUCB-MSC (Fig. 2A and B).

### Activation of TLRs and NLRs promoted osteogenic differentiation of hUCB-MSCs

**Figure 3:**
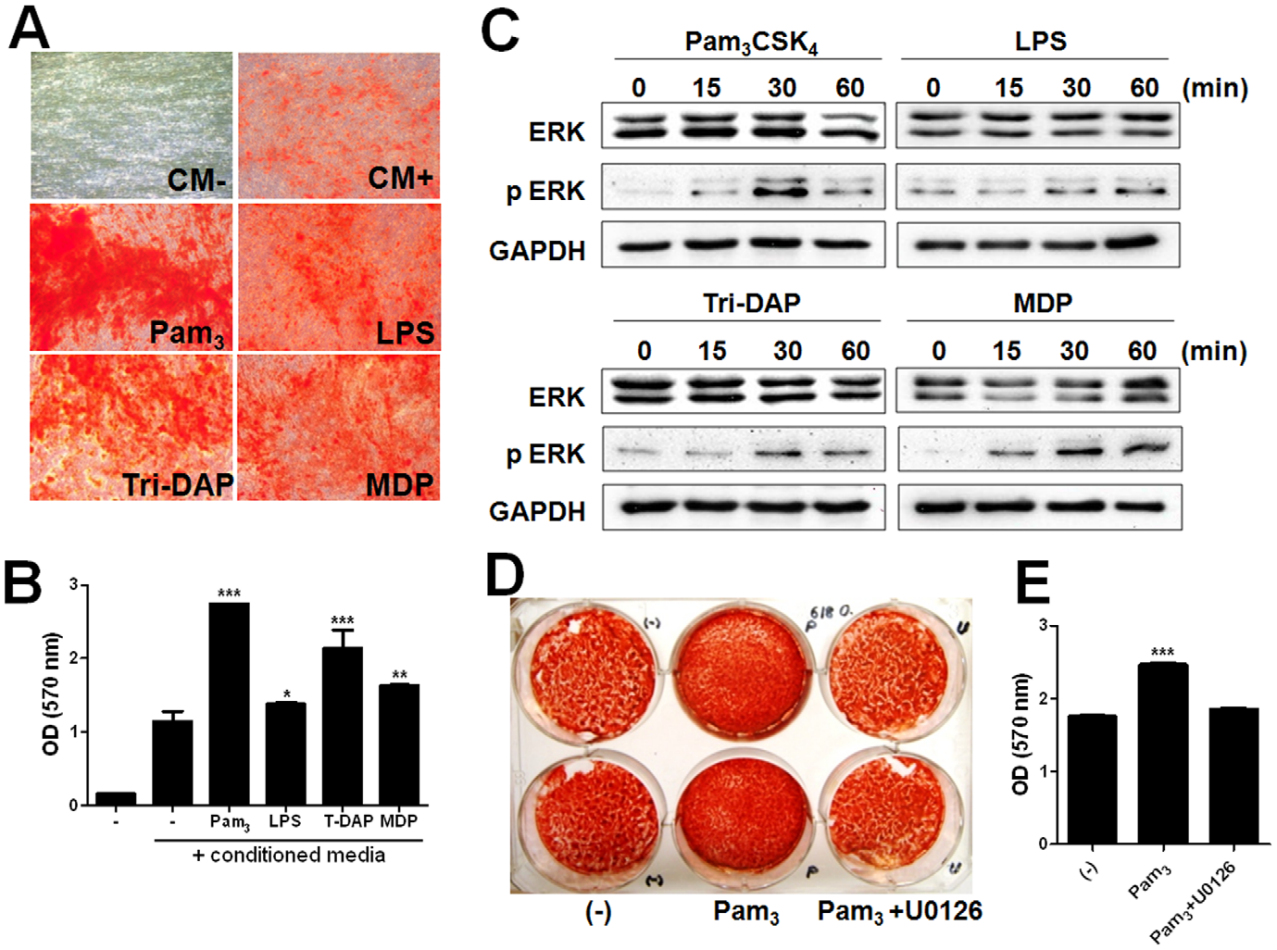
Stimulation with TLR and NLR agonists promoted osteogenic differentiation of hUCB-MSCs through phosphorylation or ERK1/2. hUCB-MSCs were grown in conditioned media at the absence or presence of Pam_3_CSK_4_, LPS, Tri-DAP, and MDP (10 µg/ml) for 2 weeks, and culture media was replaced twice per week. Osteogenesis was determined by Alizarin Red S at 2 weeks after treatment (A) and optical density was determined using ELISA at 570 nm (B). hUCB-MSCs were treated with Pam_3_CSK_4_, LPS, Tri-DAP, and MDP for 15, 30, and 60 min and ERK phosphorylation was determined by Western Blot analysis with an anti-phospho-ERK antibody (C). hUCB-MSCs were co-treated with Pam_3_CSK_4_ and U0126 for 2 weeks and determined by Alizarin Red staining (D) and quantified using ELISA at 570 nm (E). * *P*<0.05, ** *P*<0.01, *** *P*<0.001.

It has been shown that TLRs modulates the differentiation of MSCs [16], [17]. To determine whether TLRs and NLRs are involved in osteogenic differentiation of hUCB-MSCs, the cells were treated with Pam_3_CSK_4_, LPS, Tri-DAP, and MDP and cultured in standard osteogenic medium. During osteogenic differentiation of two different hUCB-MSCs (#618 and #1114), all agonists tested significantly induced higher intensity of the Alizarin red S staining (Fig. 3A and B). It has shown that extracellular signal-regulated protein kinases (ERK) activation plays an important role in the osteogenic differentiation of MSCs [22]. Therefore, we explored whether TLR and NLR agonists lead to ERK activation in hUCB-MSCs. As expected, stimulation by TLR and NLR agonists rapidly induced phosphorylation of ERK in hUCB-MSCs (Fig. 3C). To determine whether inhibition of ERK is associated with osteogenic differentiation of hUCB-MSCs, the Pam_3_CSK_4_-stimulated cells were treated with U0126 as an MEK1/2 inhibitor. In Alizarin Red S staining, treatment of U0126 restored osteogenic differentiation of hUCB-MSCs enhanced by Pam_3_CSK_4_ (Fig. 3D and E). These results indicated that both TLR and NLR signaling may promote osteogenic differentiation of hUCB-MSCs through ERK-dependent pathway.

### Tri-DAP and MDP, but not Pam_3_CSK_4_ and LPS, inhibited adipogenic differentiation of hUCB-MSCs

**Figure 4:**
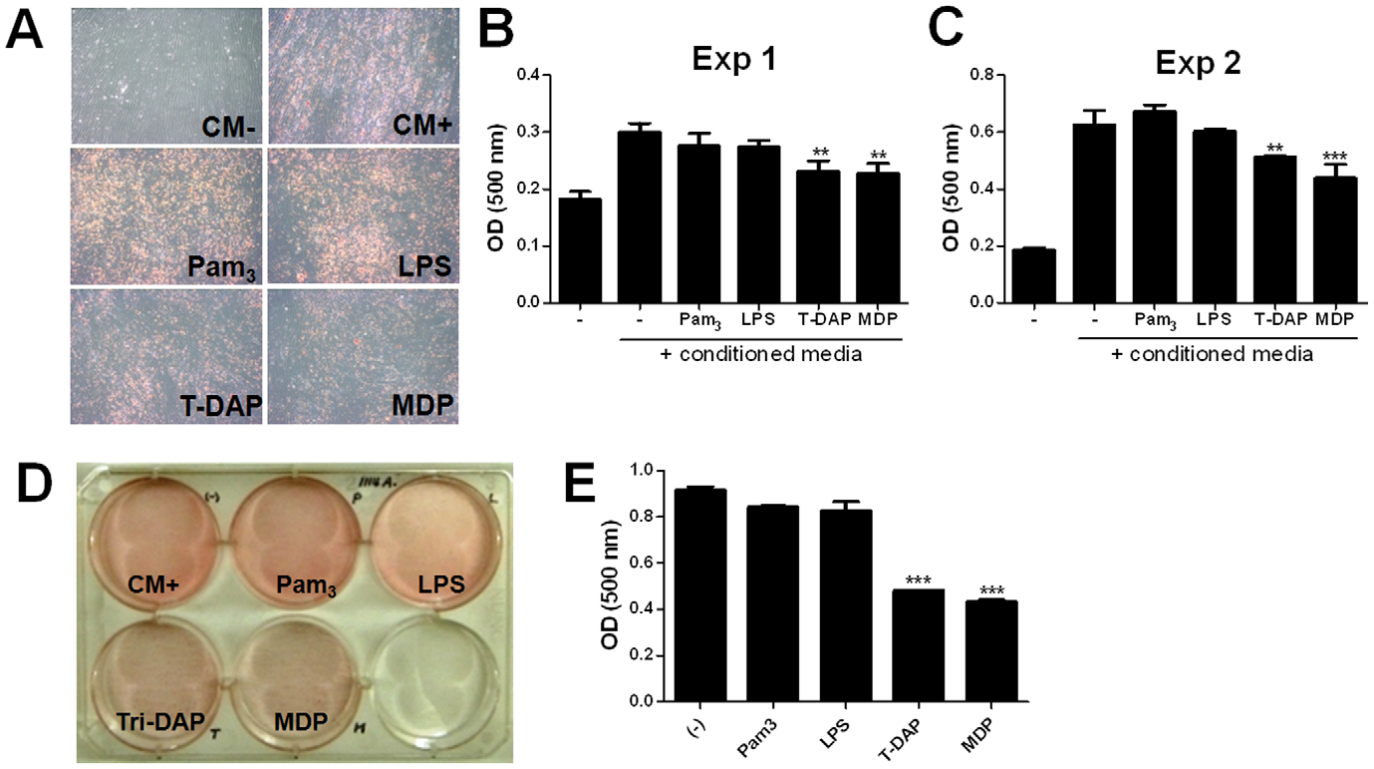
Tri-DAP and MDP inhibited adipogenic differentiation of hUCB-MSCs. hUCB-MSCs were grown in conditioned media at the absence or presence of Pam_3_CSK_4_, LPS, Tri-DAP, and MDP (10 µg/ml) for 3 weeks, and culture media was replaced twice per week. Adipogenesis was determined by Oil Red O staining (A) and level of intercellular lipid was determined using ELISA at 500 nm at 3 weeks after treatment (B and C). hUCB-MSCs (#1114) from another umbilical cord blood were grown in conditioned media at the absence or presence of each ligands for 3 weeks, and adipogenesis was determined (D) and quantified using ELISA at 500 nm (E). ** *P*<0.01, *** *P*<0.001.

To determine the effects of TLR and NLR agonists on adipogenic differentiation of hUCB-MSCs, the cells were incubated at the absence or presence of each agonist for 3 weeks. As shown in Fig. 4A–C, stimulation with Tri-DAP and MDP significantly inhibited adipogenic differentiation at 3 weeks after treatment, whereas Pam_3_CSK_4_ and LPS did not influence on adipogenic differentiation of hUCB-MSCs (Fig. 4A–C). This phenomenon was confirmed in another line of hUCB-MSCs (#1114) (Fig. 4D and E). These findings suggest that NOD1 and NOD2 signaling may be involved in adipogenic differentiation of hUCB-MSCs.

### Enhanced chondrogenic differentiation of hUCB-MSCs by TLR and NLR agonists

**Figure 5:**
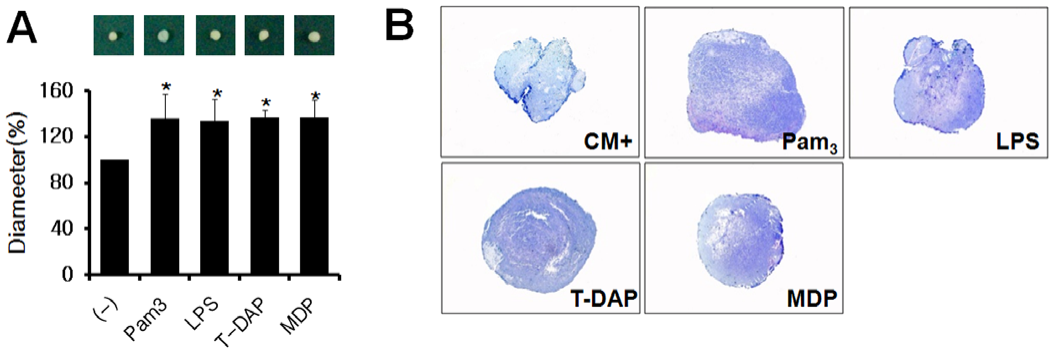
Activation of TLRs and NLRs promoted chondrogenic differentiation of hUCB-MSCs. hUCB-MSCs were prepared as pellets and they were cultured in chondrogenic medium supplemented with 500 ng/ml BMP-2 for 3 weeks. Then, the volume of pellets was measured (A) and stained with toluidine blue (B). * *P*<0.05.

To determine whether TLR and NLRs are involved in chondrogenic differentiation of hUCB-MSCs, hUCB-MSCs were maintained in BMP-2 supplemented chondrogenic medium at the absence or presence of each agonists. All TLR and NLR agonists used increased the diameter of pellets (Fig. 5A). All the pellets were positive to toluidine blue staining (Fig. 5B). These data suggest that both TLR and NLR signaling may be involved in chondrogenesis of hUCB-MSCs.

## Discussion

NLRs have been found to be involved in cytosolic recognition of microbial molecules such as PGN derivatives (by NOD1 and NOD2) and flagellin (by NLRC4/IPAF) [3]. Similarly with TLR signaling, NOD1 and NOD2 trigger inflammatory response through activation of NF-κB and MAPK. Moreover, NOD1 and NOD2 agonists, in combination with TLR agonists, synergistically induce cytokine production and activation of NF-κB and MAPK in immune cells [23], [24], [25]. These findings suggest that NLR signaling may be closely related in TLR-mediated events. As TLRs modulate the functions of a variety of MSCs, in this study, we explored the role of NLRs, particularly NOD1 and NOD2, on hUCB-MSCs function. In addition, because most studies about the role of TLRs have been performed in BM- or adipose tissue-derived MSCs, we also confirmed the effect of TLR2 and TLR4 on UCB-MSC functions.

A previous study showed that cord blood MSCs expressed low levels of TLR1, 3, 5, 9 and high level of TLR4 [19]. However, in our study, both TLR2 and TLR4 were expressed in hUCB-MSCs, although TLR4 expression was much stronger than TLR2. This discrepancy might be due to difference of PCR condition used or MSCs origin. Many studies also showed functional expression of TLR2 in BM- or adipose tissue-derived MSCs (ASC) [16], [17], [18], [26]. Moreover, in this study, Pam_3_CSK_4_ and LPS stimulation led to IL-8 production in UCB-MSC, suggesting that hUCB-MSCs functionally express TLR2 and TLR4.

It has been known that NOD1 is ubiquitously expressed in a variety of cell types, whereas NOD2 is mainly expressed in immune cells [27]. In this study, the genes of NOD1 and NOD2 were apparently expressed in hUCB-MSCs, although expression levels were relatively weaker than those of TLR2 and TLR4. In addition, mRNA of Rip2/RICK, an adaptor molecule of NOD1 and NOD2, was also expressed. Stimulation with Tri-DAP and MDP, NOD1 and NOD2 agonists, led to IL-8 production, indicating the functional expression of NOD1 and NOD2 in hUCB-MSCs.

There are discrepancies about the effect of TLRs on the MSC proliferation. A recent study showed that TLR agonists including PGN, LPS, poly I:C, and flagellin did not affect the proliferation of human ASC (hASC) [16]. Only CpG-ODN, TLR9 agonists, reduced slightly hASC proliferation [16]. This was confirmed by a study of Lombardo *et al*. [26], showing that TLR3 and TLR4 agonists had no effect on the proliferation of hASCs. In contrast, downregulation of MyD88, a common adaptor molecule for TLRs except TLR3, using siRNA inhibited the proliferation of hASC [21]. In addition, TLR agonists such as Pam3Cys and LPS enhanced the proliferation of mouse BM-MSCs [17], [20], suggesting involvement of TLRs on MSC proliferation. Collectively, these results indicate that the effect of TLRs on MSC proliferation may be a cell-type specific event. In our study, the agonists of NOD1 and NOD2 as well as TLR2 and TLR4 did not influence hUCB-MSCs proliferation. It is needed to clarify the effect of NOD1 and NOD2 on the proliferation of different types of MSCs.

It has been well known that TLRs are associated with MSC differentiation. Particularly, TLRs seem to be involved in osteogenic rather than adipogenic differentiation of MSC. TLR3 and TLR4 agonists significantly increased osteogenic differentiation of hASCs, but did not affect adipogenic differentiation [21], [26]. Other TLR agonists (poly I:C, flagellin, CpG-ODN) also did not influence adipogenic differentiation of hASC [16]. Interestingly, PGN inhibited adipogenic differentiation of hASCs significantly [16], [21]. In those studies, PGN was used as TLR2 agonist. However, a previous study by Travassos *et al.* [28] revealed that highly purified PGN was not detected by TLR2. They suggested that cell wall contaminants such as lipoteichoic acid (LTA) or lipoproteins present in PGN preparations are responsible for TLR2-dependent cell activation [28]. In our study, Tri-DAP and MDP significantly inhibited adipogenic differentiation of hUCB-MSCs at 2 and 4 weeks after stimulation, whereas Pam_3_CSK_4_ and LPS did not, suggesting involvement of NOD1 and NOD2 on adipogenic differentiation of hUCB-MSCs. As well known, NOD1 and NOD2 recognize PGN derivatives, *meso*-DAP and MDP, respectively. Accordingly, it is likely that NOD1 and NOD2 are involved in PGN-mediated inhibition of adipogenic differentiation of hASCs. It should be clarified the role of TLR2 on adipogenic differentiation of MSC and which receptor originally mediates inhibitory effect of PGN. In addition, various TLR agonists enhance osteogenic differentiation of MSCs [16], [21], [26]. Our results showed that all TLR and NLR agonists used increased osteogenic differentiation of hUCB-MSCs, suggesting that NLRs as well as TLRs may be involved in osteogenic differentiation of MSCs. ERK phosphorylation is correlated with the osteogenic differentiation of MSCs [22], [29]. Because TLR and NLR agonists enhanced osteogenic differentiation of hUCB-MSCs, we examined ERK phosphorylation by the agonists. LPS and MDP induced ERK phosphorylation within 1 h in mouse macrophages [24], [30]. In this study, Pam_3_CSK_4_ and MDP induced ERK phosphorylation in hUCB-MSCs from 15 min after treatment and the level reached to peak at 30 min post-treatment. LPS and Tri-DAP induced ERK phosphorylation from 30 min post-treatment. ERK was strongly phosphorylated in the hUCB-MSCs treated with Pam_3_CSK_4_, whereas LPS induced only mild activation of ERK, which correlated with intensity of Alizarin Red S staining. Moreover, U0126, MEK1/2 inhibitor, inhibited osteogenic differentiation enhanced by Pam_3_CSK_4_. Taken together, it is likely that ERK signaling is critical for osteogenic differentiation of hUCB-MSCs induced by TLR and NLR stimulation.

In our knowledge, there is no report about the effect of TLRs on chondrogenic differentiation of MSCs, except the study by Pevsner-Fischer *et al* [17]. They revealed that TLR2 stimulation with Pam_3_Cys inhibited induced differentiation of MSCs into osteogenic, adipogenic, or chondrogenic lineages. However, in this study, all used agonists including Pam_3_CSK_4_ enhanced the chondrogenic differentiation potential of hUCB-MSCs. The reason for this discrepancy remains to be elucidated.

In conclusion, the present study revealed novel information that NOD1 and NOD2 as well as TLRs are involved in regulating the differentiation of hUCB-MSCs. These findings are expected to provide better understanding of the biological function of MSCs. Further study using in vivo model is need to clarify physiological role of NOD1 and NOD2 on MSC functions.
